# Validating a new generation filter system for visualizing 5-ALA-induced PpIX fluorescence in malignant glioma surgery: a proof of principle study

**DOI:** 10.1007/s00701-020-04227-7

**Published:** 2020-02-07

**Authors:** Eric Suero Molina, Louise Stögbauer, Astrid Jeibmann, Nils Warneke, Walter Stummer

**Affiliations:** 1 Department of Neurosurgery, University Hospital of Münster, Albert-Schweitzer-Campus 1, A1, D-48149 Münster, Germany; 2 Institute of Neuropathology, University Hospital of Münster, Münster, Germany

**Keywords:** 5-ALA, Fluorescence guidance, Malignant glioma

## Abstract

**Background:**

The BLUE 400 filter system (Carl Zeiss Meditec, Oberkochen, Germany) has provided visualization of 5-ALA-induced fluorescence-guided surgery for more than 20 years. Nevertheless, constraints, e.g., limited background discrimination during hemostasis, obstruct fluency of surgery. A novel filter with improved background visualization was developed, requiring validation regarding fluorescence discrimination. The aim of this article is to determine diagnostic accuracy and perception of protoporphyrin IX (PpIX) discrimination of a novel filter system with higher background illumination (BLUE 400 AR) compared with the gold standard, BLUE 400.

**Methods:**

A surgical microscope equipped with both BLUE 400 and BLUE 400 AR was used. Comparisons were performed on a biological basis and on the visual perception of margins. High-resolution images were compared during and after surgery by senior neurosurgeons. In a predefined biopsy algorithm, four biopsies per patient at tumor margins of PpIX fluorescence and adjacent brain were acquired using BLUE 400 AR only from regions intended for resection and assessed for cell count and density.

**Results:**

Thirty-two patients with malignant gliomas were included in this study. BLUE 400 AR markedly enhanced the brightness of the surgical field, allowing superior discrimination of brain anatomy. A total of 128 biopsies from fluorescence margins were collected. Positive predictive value (PPV) was 98.44% (95% CI, 90.06–99.77%) for malignant glioma. Residual median cell density in non-fluorescent tissue was 13% (IQR 13 to 31). Perception of the location of fluorescent margins on HD images was equivalent for both filter combinations.

**Conclusions:**

BLUE 400 AR demonstrated superior background compared with conventional BLUE 400 in malignant glioma surgery but comparable fluorescence margins and PPV. Therefore, BLUE 400 AR can be considered safe and effective in supporting malignant glioma surgery.

**Electronic supplementary material:**

The online version of this article (10.1007/s00701-020-04227-7) contains supplementary material, which is available to authorized users.

## Introduction

Fluorescence-guided surgery (FGS) with 5-aminolevulinic acid (5-ALA) is well established in malignant glioma surgery. After exogenous administration, 5-ALA elicits synthesis and accumulation of endogenous fluorescent protoporphyrin IX (PpIX) in malignant gliomas [5, 8, 11, 14]. First introduced in 1998 [10–12], and corroborated for efficacy and safety by a randomized phase III trial in 2006 [9], 5-ALA was approved by the European Medicines Agency and, more recently, by the FDA in the USA.

With this method, fluorescence is assessed visually using special optical filters, which have remained literally unchanged since their inception in 1998 [10]. Fluorescence appears as violet-red on a blue background, the background resulting from a minor portion of the remitted blue excitation light combined with autofluorescence [10]. To date, surgeons experience a more or less frequent need for changing to white light for reorientation and/or hemostasis. This interrupts the fluency of surgery and results in sometimes prolonged periods of adaption [13].

As a first attempt to improve the surgical setting during FGS, we recently combined 5-ALA with fluorescein [15, 17] for improved background illumination of the surgical field. Fluorescein circulates in perfused tissues, giving a high level of fluorescence in normal brain [4, 6, 7]. Others are using hyperspectral [1] or fluorescence lifetime imaging [2] for highlighting regions of fluorescence using augmented reality overlays. The present technique incorporates a novel filter technology for visual fluorescence discrimination with a brighter background image, BLUE 400 AR.

However, 5-ALA induced porphyrins were initially approved using the traditional filter combinations which showed a particular fluorescence distribution but do not capture cells with small amounts of porphyrins in the low-density infiltration zone of gliomas. Such remnants can be detected using spectrography [14, 18, 19]. To be equally safe and effective, any new technology would have to be able to discriminate a comparable amount of visually detectable fluorescence and associated cells to a comparable cell density. If discrimination was inferior, efficacy would be impaired compared with the original method. If, on the other hand, the sensitivity of a method was greater, this could potentially result in more extensive resections with implications for surgical safety. For instance, spectrography [18, 19] has a higher sensitivity but lower specificity and positive predictive value, leading to more false positive interpretations of tissue being tumor.

Our main concern with the new method was that the greater background brightness of BLUE 400 AR would result in less sensitivity or a loss of contrast at margins of fluorescence. This concern was the basis for the evaluations in the present study.

In order to corroborate comparability of the new system, some form of validation was required. We chose a two-fold approach, first to determine the drop in cell density at the margins using the new method while adhering to a predefined biopsy algorithm, as well as measures of diagnostic accuracy, in a fashion comparable to the previous study [14], furthermore, to visually compare margins as perceived by surgeons. Thus, our approach tests biological efficacy of the novel method and comparability of perception rather than measuring purely technical characteristics of the new hardware.

## Methods

### BLUE 400 AR filter system

A novel filter module (BLUE 400 AR, Carl Zeiss Meditec, Oberkochen, Germany) was designed to visualize PpIX fluorescence with optimized background illumination. The module was integrated in an OPMI PENTERO 900 microscope with CE certification for patient use and could be toggled back and forth to the BLUE 400 mode or to normal white light illumination. The microscope was equipped with a 3-CMOS full high-definition camera (1080p resolution).

With the conventional BLUE 400 observation filter, the complete PpIX fluorescence spectrum and a part of the blue-violet excitation light (400–410 nm) are transmitted to the surgeon’s eyepiece. This enables tissue discrimination of red PpIX fluorescence on a blue background [10].

**Fig. 1:**
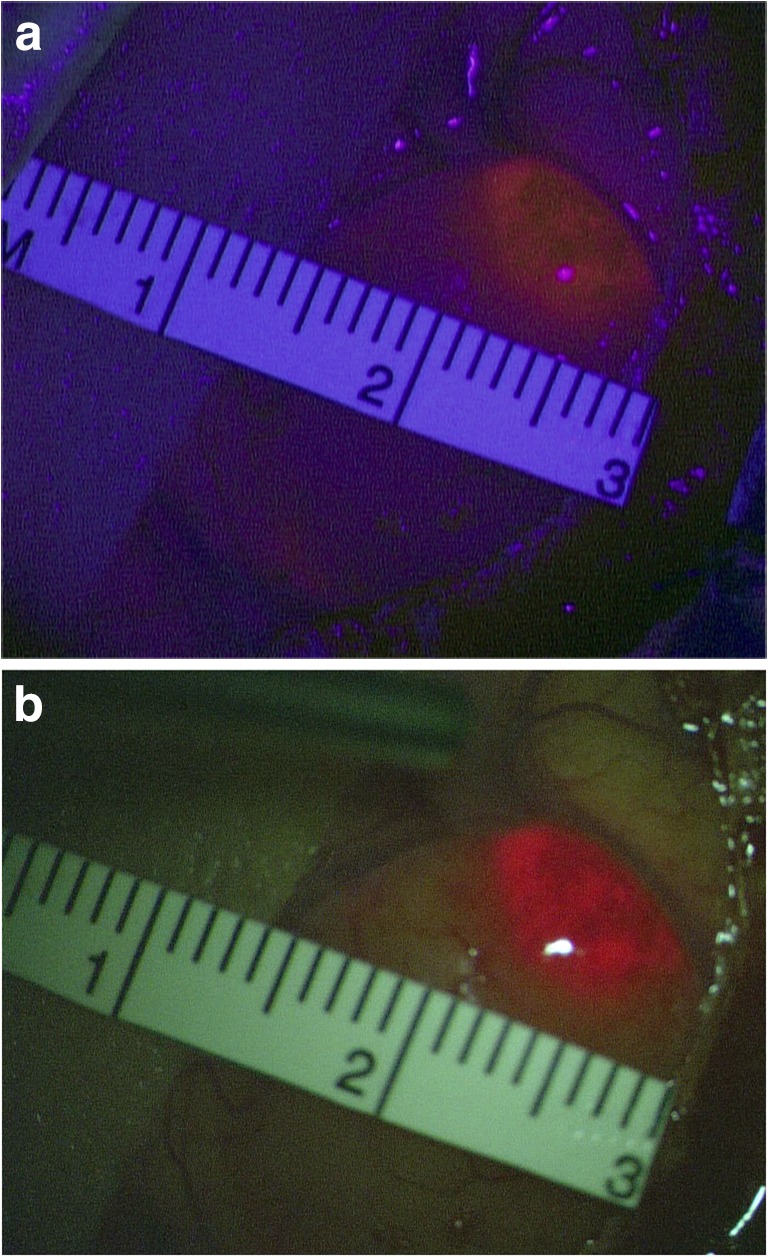
Intraoperative microscopic view of a superficial glioblastoma under **a** BLUE 400 conventional filter and **b** filter BLUE 400 AR. Fine cortical vessels are well depicted under the novel filter system, as well as sulci and adjacent brain tissue

The BLUE 400 AR filter features the same excitation in the range of 400-410 nm but allows an expanded emission wavelength (600–710 nm) to be observed by the surgeon. However, it also includes other discrete spectral components to allow better background visualization. The filter combination thereby enables the surgeon to perceive more background detail, with the non-fluorescing brain appearing yellow-green (Fig. 1, Video), while tumor fluorescence has a more orange shade.

The BLUE 400 AR and the BLUE 400 filter set have similar effective excitation and observation spectra with respect to PpIX. In order to visualize the non-fluorescent tissue, a small amount of the excitation spectrum—the so-called ambient illumination—can be observed simultaneously with the PpIX fluorescence.

For the BLUE 400 filter set, a defined amount of blue light is used and the non-fluorescent tissue is perceived in blue color. However, for the BLUE 400 AR filter set, amounts of blue and orange light are used for the ambient illumination. To ensure an almost natural appearance of non-fluorescent tissue, the amounts of light are specifically balanced so that the white point and the balance point of the BLUE 400 AR filter set are as close as possible. As a result, the non-fluorescent tissue can be observed in a more natural color than with BLUE 400. In both cases, the ambient illumination has a similar brightness and does not overlap with the PpIX emission spectrum.

### Patient collective and general procedures

This was a prospective, single-center analysis of patients with suspected malignant gliomas operated at our institution between June 2017 and January 2018. The evaluation was approved by the ethical committee of the University of Münster and followed the principles outlined in the Declaration of Helsinki. Patient informed consent was acquired.

All patients were pretreated with dexamethasone (3 × 4 mg). 5-ALA (Gliolan®, Wedel, Germany) was administered 3–4 h orally prior to induction of anesthesia. Surgery was performed using standard techniques including neuronavigation, ultrasound, and, in selected patients, awake craniotomy techniques. After surgery, patients were maintained in darkened surroundings (no direct artificial lighting, no daylight) for 24 h after administration.

In all patients, resection was performed as usual. However, when tumor borders were identified which were intended for resection, the microscope was first switched to BLUE 400 AR. When useful fluorescence borders were observed, the surgical ruler was superimposed for image acquisition, and biopsies collected as described below, based on the BLUE 400 AR signal. Surgical decisions were only made using BLUE 400. During surgery and fluorescence visualization, microscope working distance was maintained at around 25 cm from the operating field and light intensity was set to 100% for both fluorescence modalities.

### Images assessment

For correlating margins of PpIX fluorescence using the new and the traditional filters, high-definition images of two corresponding surgical locations were acquired with each system (Fig. 1). The images had to clearly visualize at least two areas of transition between fluorescing and non-fluorescing tissue. During surgery, the transition zones were superimposed with a surgical ruler for later analysis. A total of 17 pairs of images were acquired in the course of the study, randomly mixed, and demonstrated to 10 senior surgeons on high-definition computer screens for assessing their individual perception of fluorescence margins on a video screen relative to the units of the surgical ruler. Care was taken not to present comparable images in close succession. These numbers were recorded and averaged.

For every border, the unit ascribed to the border from the ruler of BLUE 400 was subtracted from the value derived for BLUE 400 AR to give a calculated difference in mm between the respective borders.

#### Statistical considerations

The primary aim of this study was to estimate the positive predictive value of 5-ALA induced-fluorescence using the new method in samples taken from the border of the fluorescent regions. Therefore, statistical analysis consisted of data description rather than testing of any pre-specified statistical hypothesis system.

Nevertheless, we closely adhered to the sample size in a previous study in order to compare measures of diagnostic accuracy and related 95% confidence intervals. Non-overlapping 95% confidence intervals signify an error probability of at least *p* < 0.05.

Box-Whisker diagrams were created for illustrating distributions. For exploratory statistical comparison, a non-parametric Friedman test was applied for exploring global homogeneity followed by Wilcoxon’s tests for pairwise comparisons of dependent observations (Wilcoxon signed-rank tests).

The positive predictive value (PPV) of tissue fluorescence at the biopsy level was defined as the percentage of biopsies allowing positive tumor cell identification in all biopsies taken from the sites with fluorescence; the negative predictive value the percentage of non-fluorescing samples without fluorescence tumor cells; the sensitivity the percentage of fluorescing samples with tumor cells, relative to all biopsies containing tumor cells with or without fluorescence; and the specificity as the number of non-fluorescing samples without tumor cells relative to all samples, with or without fluorescence but without tumor cells. The point estimates and their exact 95% confidence interval were calculated for each measure.

Statistical analyses included univariate analyses performed with commercially available software (IBM SPSS, Version 25).

#### Biopsy collection and evaluation

From each patient, strictly four biopsies each were obtained at the margins between fluorescent (2 biopsies) and non-fluorescent tissue (2 biopsies), as defined using BLUE 400 AR, within a distance of 2 mm from the border in an adaption of the methodology previously described [14], after resection based on the BLUE 400 image. In contradistinction to the earlier protocol, no samples were collected from the main fluorescing tumor mass or in normally appearing brain remote from the fluorescing tumor mass.

Histopathological evaluation was carried out by blinded neuropathologist (Institute of Neuropathology, University Hospital of Münster) according to the present WHO grading system in hematoxylin and eosin (HE) and elastica van Gieson stained samples. Immunohistochemical and molecular analysis of IDH-1 mutations, Ki-67 MIB1 proliferation index, glial fibrillary acidic protein (GFAP), and MGMT methylation were also performed.

Tumor cell density was determined in HE-stained tissue. Images for counting were taken using 10-fold magnification. A representative area for quantitative counting of tumor cells was selected. On average, digital counting was performed on an area of at least 70,000,000 μm^2^, which was finally extrapolated to an area of x cells per cm^2^ for a simpler demonstration of results. We also evaluated tumor cell density as previously reported [14], using a semi-quantitative scale comprising five categories: 0%, 1–25%, 26–50%, 51–75%, and 76–100%, of which average values, i.e., 0%, 13%, 38%, 63%, and 88% respectively, were used for statistical calculations. Furthermore, biopsies were categorized as solidly proliferating tumor tissue, infiltrating tumor, necrosis, or reactive brain tissue.

## Results

A total of thirty-two patients were included (male to female ratio 14:18, median age was 63 years, range 34–84), of which 25 (78%) harbored a glioblastoma (WHO °IV) and 7 (22%) an anaplastic astrocytoma (WHO °III; Table 1). Furthermore, a total of 128 biopsy specimens from the fluorescent (*n* = 64) and non-fluorescent infiltration tumor margins were acquired (*n* = 64) (Table 1). No adverse events were recorded. Complete resection of enhancing tumor (CRET) in patients with ex ante planned complete resection was achieved in all patients (21 Patients; 100%) In 11 patients, a subtotal resection was achieved either due to eloquent location (*n* = 10) or to avoid injury of perforator vessels (*n* = 1), e.g., in an insular tumor. Mean progression-free survival was 8 months (± SD 5 months, median progression-free survival not reached), and mean overall survival time was 11.2 months (± SD 7.6, median overall survival not reached). Median observation time was 12 months (95% CI 9.120–14.756), with 91% of patients surviving at this point of time.

**Table 1:** Patient demographics

Demographics of patients population	
Sex (*n*)	
Male	14
Female	18
Age (years)	
Median	63
Range	34–84
Histology (*n*)	
Anaplastic astrocytoma (WHO °III)	7
Glioblastoma (WHO °IV)	25
Recurrence	
Yes	11
No	21
PFS (months)	
Mean (SD)*	8 (± 5)
Overall survival (months)	
Survival at median observation time	91%
Mean (SD)*	11.2 (± 7.6)
Observation time (months)	
Median	12
95% CI	9.12–14.7

PFS, progression-free survival

*Median progression-free and overall survival not reached

### Descriptive fluorescence visualization

The BLUE 400 AR filter provided superior background illumination compared with BLUE 400 (Fig. 1, Video). Hemostasis could be sufficiently achieved under the fluorescent light, without the need to alternate between the fluorescence filter and white-light microscopy.

### Cell count and density

**Fig. 2:**
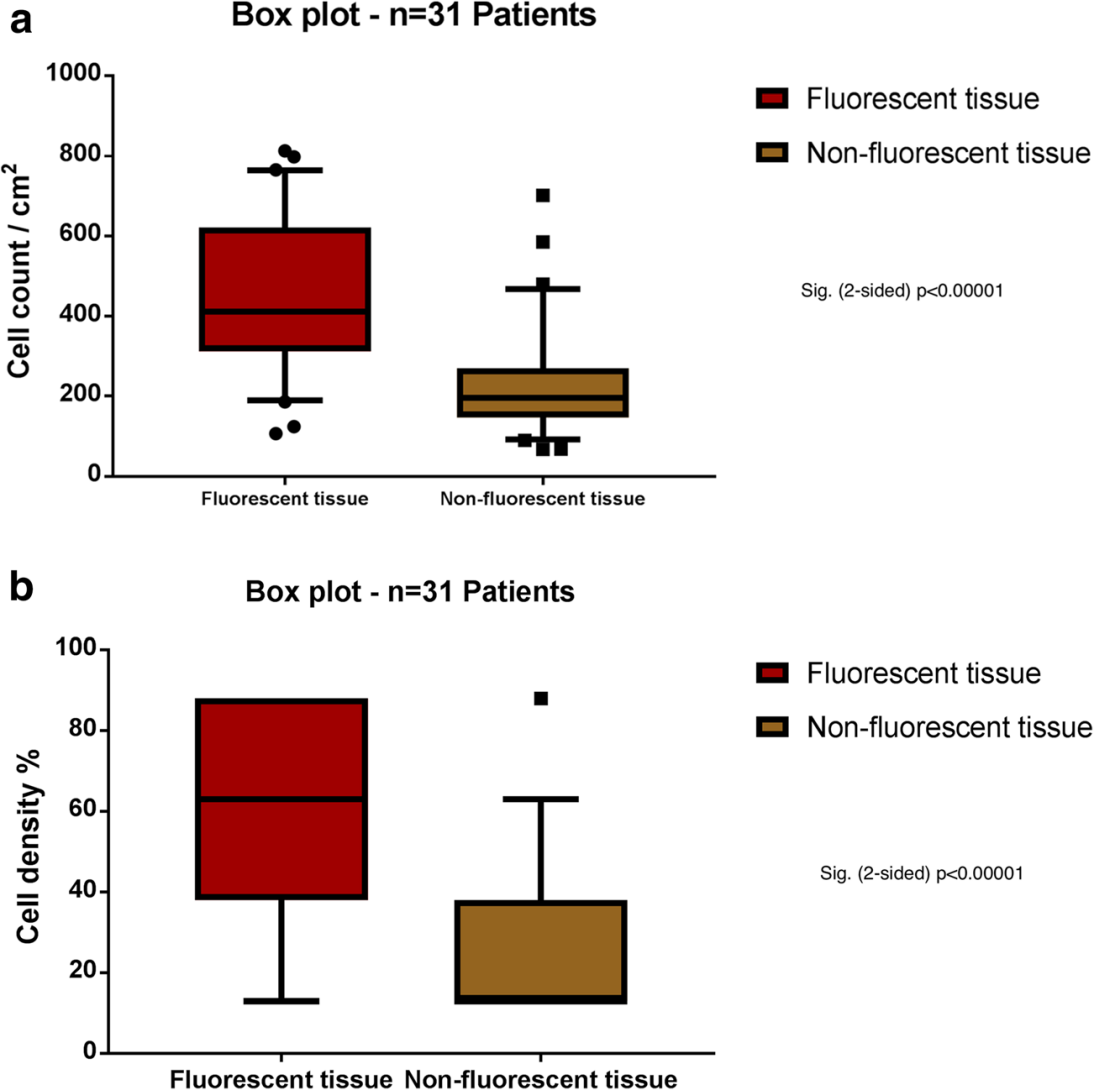
Box plot analyses comparing **a** cell count/cm^2^ and **b** tumor cell density between fluorescent and non-fluorescent biopsies. Both demonstrated a highly significant difference between the compared groups (*p* < 0.0001)

Cell count was performed in 128 biopsies from all 32 patients observed under BLUE 400 AR. Median cell count was 411 cells/cm^2^ (range 107–813) in fluorescent biopsies, and 195 cells/cm^2^ (range 67–702) in non-fluorescent biopsies (*p* < 0.0001, Fig. 2a).

**Fig. 3:**
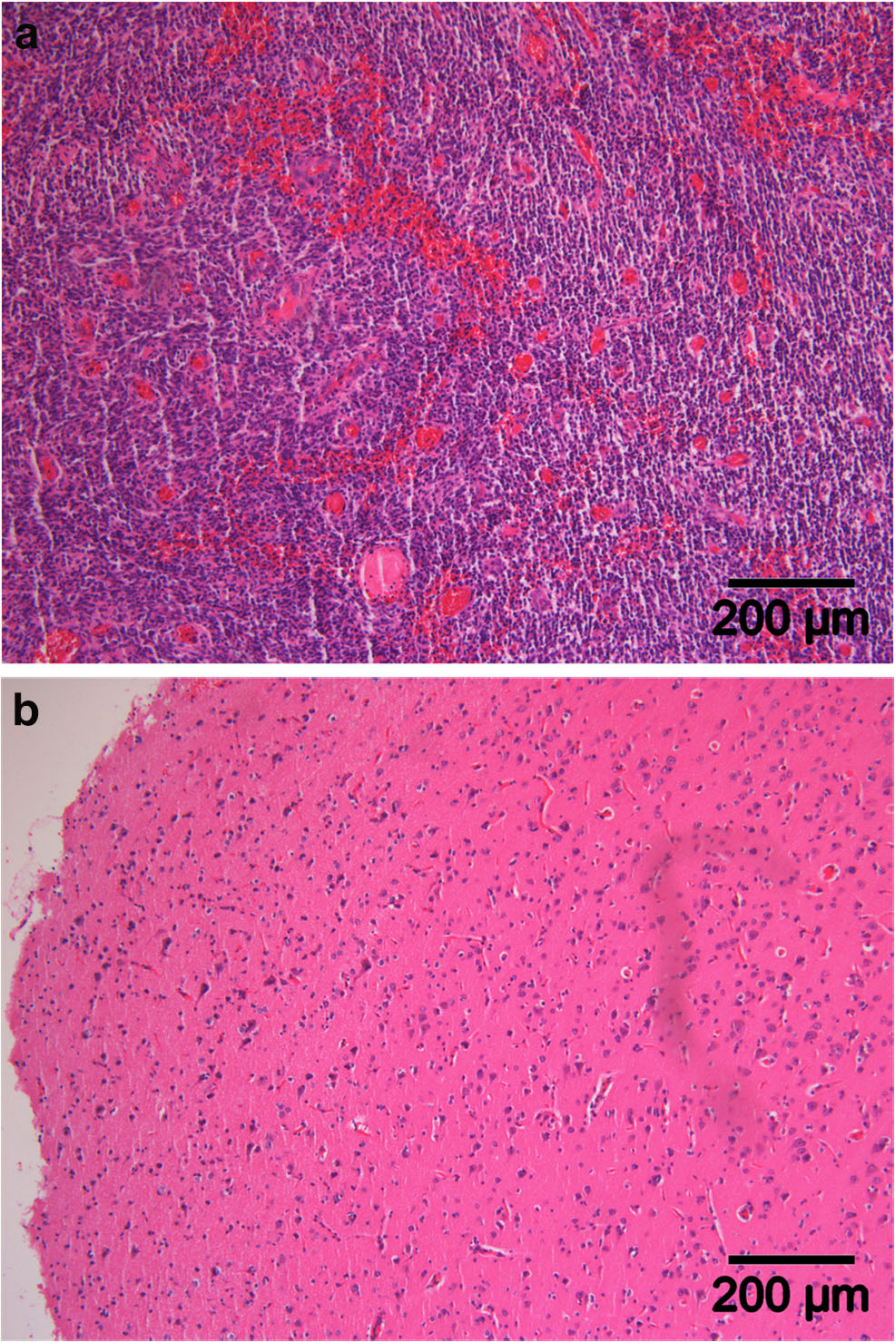
Microscopic view of **a** fluorescent and **b** non-fluorescent tissue demonstrating the high and low cellularity within these tumor regions

On the semi-quantitative scale, median cell density in fluorescent marginal tissue was 63%, inter-quartile range 38 to 88 (mean 57.35% ± 24.75 SD), whereas non-fluorescent marginal tissue showed a median tumor cell density of 13% (inter-quartile range 13 to 38; mean 23.48% ± 19.12 SD) (*p* < 0.0001, Figs. 2b and 3).

**Fig. 4:**
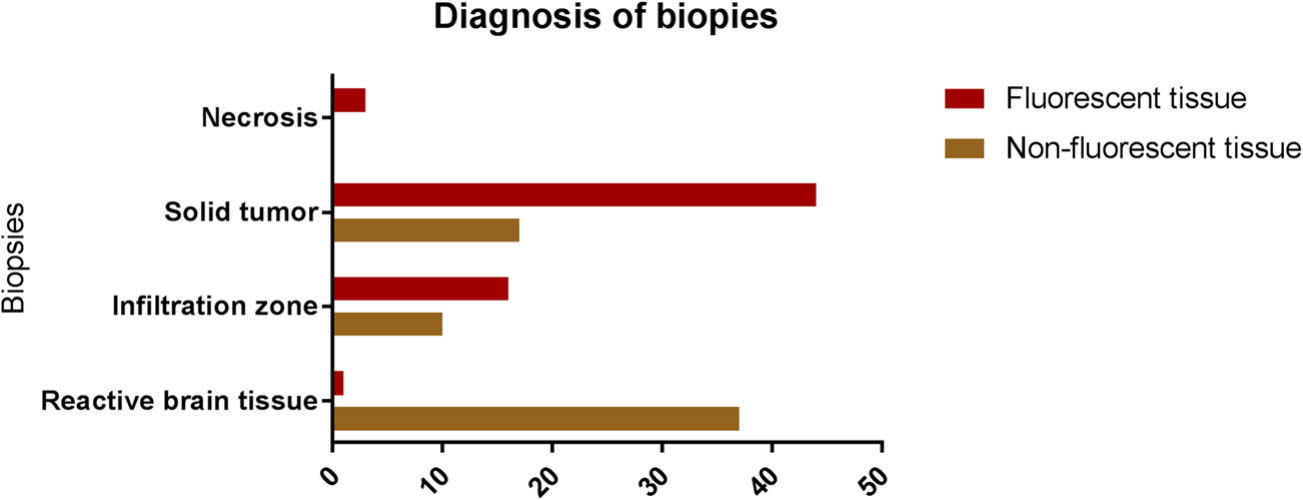
Description of diagnosis of biopsies among the analyzed specimens (*n* = 128), from both fluorescent (*n* = 64) and non-fluorescent tumor infiltration zones (*n* = 64). Note that only one case of reactive brain tissue could be found within biopsies acquired from the fluorescent tumor tissue observed under the novel filter BLUE 400 AR

Qualitatively, biopsies from fluorescent tissue (*n* = 64, Fig. 4) demonstrated tumor tissue (*n* = 44), infiltrating tumor zone (*n* = 16), necrosis (*n* = 3) and reactive brain tissue (n = 1), whereas biopsies in non-fluorescent tumor infiltration zone (n = 64) showed tumor tissue (*n* = 17), infiltrating tumor (*n* = 10), and reactive brain tissue (*n* = 37) (Fig. 3b).

### Sensitivity, specificity, positive and negative predictive values

**Fig. 5:**
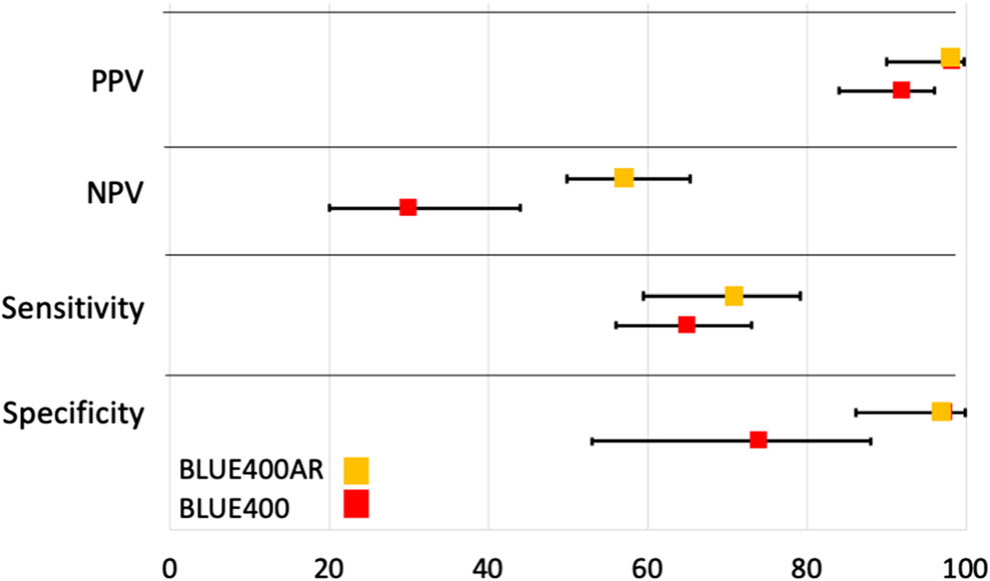
Comparison of acquired data with BLUE 400 AR during this study with older data generated for BLUE 400 [14], demonstrating an overall good correspondence regarding PPV and specificity when comparing means with their corresponding 95% CI. Furthermore, NPV and hereto-related sensitivity appeared higher

Sensitivity of fluorescence to detect tumor tissue was 70.89% (95% CI 59.43–79.21%) and specificity 97.37% (95% CI 86.19–99.93%). The PPV was 98.44% (95% CI 90.06–99.77%), whereas the negative predictive value was 57.81% (95% CI 49.88–65.36%). Only one biopsy in the fluorescent tissue could not be diagnosed as tumor (1/64) in a patient with glioblastoma. We compared the present results to older data generated for BLUE 400 [14] (Fig. 5). We found good overlap regarding PPV and specificity when comparing means with their corresponding 95% CI; however, the NPV and related to this, the sensitivity, appeared higher. For NPV, the 95% CI did not overlap, suggesting that NPV might in fact be slightly superior with the novel method (Fig. 5).

#### Image assessment

**Fig. 6:**
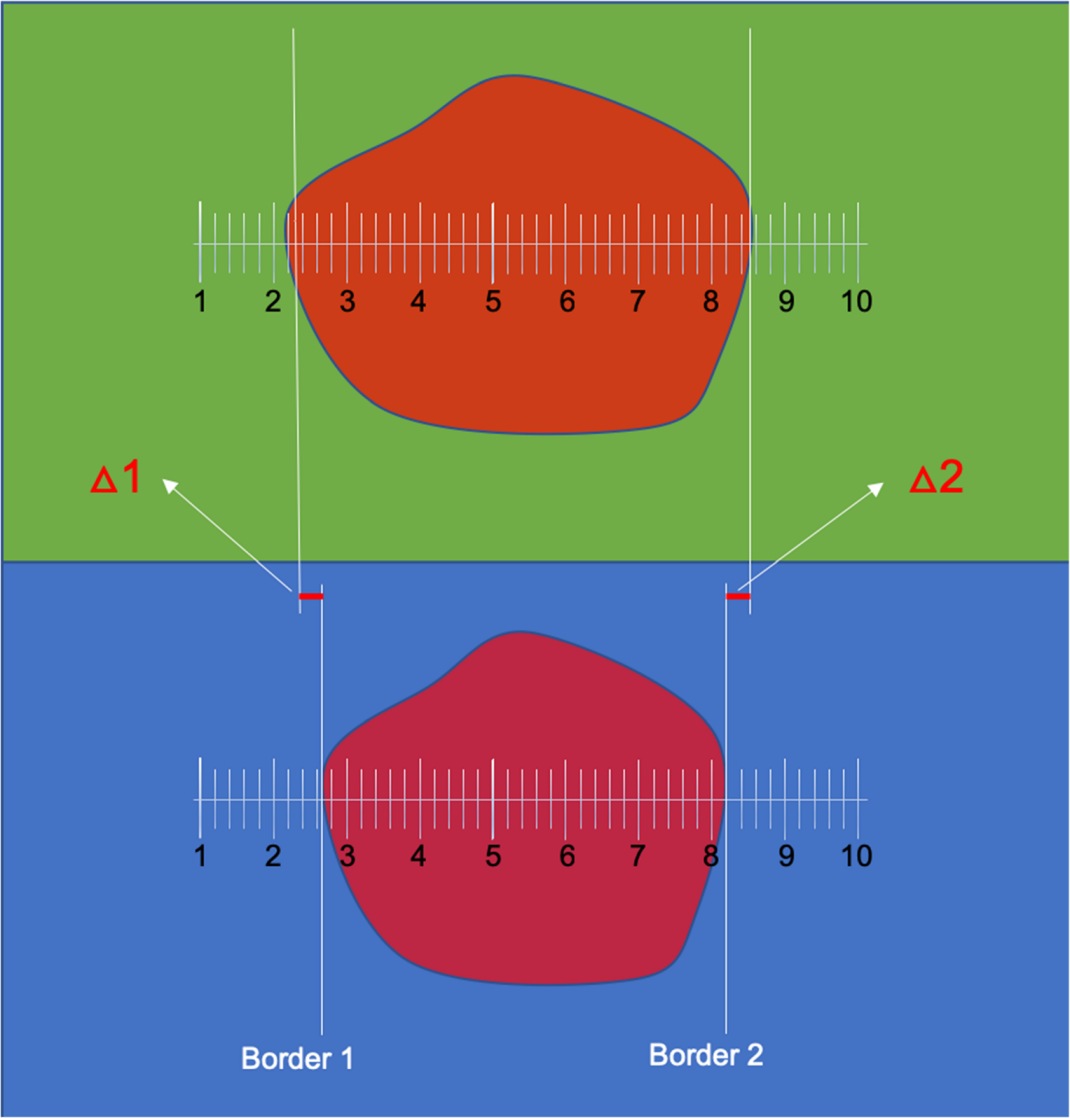
Scheme demonstrating method for visually comparing fluorescence borders using images taken respectively under BLUE 400 and BLUE 400 AR modalities

Ten surgeons reviewed 17 images displaying the same surgical field visualized using both different fluorescence moieties, with 2 borders each along the axis of the ruler. This gave a total of 340 comparisons (Figs. 1 and 6). There were no significant differences between fluorescence margins (Table 2). Means and median differences were the same.

**Table 2:** Differences between measured distances at the tumor margins in 17 different comparative images of the surgical field under both BLUE 400 and BLUE 400 AR filter system performed by 10 different experienced neurosurgeons. There was no statistical difference between the perceived fluorescent tumor margin under the BLUE 400 and the BLUE 400 AR filter system

*N* = 170	Border 1 (mm)	Border 2 (mm)
Mean	0.03	0.04
Median	0	0.1
Minimum	− 0.38	− 2.38
Maximum	0.75	1.13
95% CI	− 0.0016–0.0553	− 0.02–0.09

## Discussion

With this study, we attempted to validate a new filter technology by comparing a novel technology to an older established technology which has been the basis for 5-ALA FGR for the last 20 years. We found the new filters to offer improved background definition, enabling a better understanding of anatomy and allowing manipulations of non-fluorescing tissue, e.g., coagulation, normally not possible with the older technique, as illustrated in Fig. 1 and Video 1. We do concede that any image viewed for documentation is only as good as video chain that are being recorded or viewed with. We consider the original visual image available through the microscope superior to the images provided in this manuscript, which were acquired with either BLUE 400 or BLUE 4000 AR cameras.

We noted the visual perception of fluorescent borders was to be highly comparable to the older technology. We also reproduced a significant drop in tumor cell density by resecting fluorescent tissue, to about 12% (median) in marginal, non-fluorescent tissue that has previously been established for BLUE 400 [14], a similar PPV 98% and specificity, which we calculated using our specific sampling regime. On a by note, fluorescence has been shown to extend beyond contrast-enhancing tumor [8, 16] in many cases and our observations again confirm that in over half of biopsies in non-fluorescing tissue residual tumor, cells can be detected, albeit at a low density.

We believe a high PPV, determined at the very margin of fluorescence, and the demonstration of a clear decline in tumor cell density in non-fluorescing tissue not destined to be removed are the two key determinants which characterize fluorescence in these diffusely infiltrating tumors. The surgeon should be confident that what is fluorescing at the tumor margin in fact contains tumor cells, and this fluorescence will take him to the margins of what he wants to resect.

We did however note that the NPV appeared higher than previously reported [14], which could be interpreted as showing superiority of BLUE 400 AR over BLUE 400. Another explanation may simply be variations in the interpretation of histological images by a given pathologist, specifically cell density when we used our semi-quantitative assessment scheme. This factor is a possible confounder of a biologically based validation system for different compounds and hardware systems.

Apart from the variability imposed by raters regarding neuropathological assessments, PPV and other measures of diagnostic accuracy are subject to multiple other biases and confounders. For instance, in diffusely invading tumors, PPV and NPV depend strongly on where the samples are taken in relation to the tumor mass. NPV will be lower at the immediate non-fluorescing margin where infiltrating tumor cells will have a higher concentration and will increase further the samples are taken away from the main tumor mass. PPV will be very high in the center of the tumor, rather than at the margins, if the method is not unerringly selective. The measures “sensitivity” and “specificity” both contain information from samples taken from fluorescing and non-fluorescing tissues and will similarly depend on the location of biopsies.

Together, all calculation of measures of diagnostic accuracy will depend on the number of samples taken per patient and region. For this reason, we have adhered to a strict biopsy regimen in this study as in earlier work [14] to enable a certain confidence for directly comparing methods. Similar thoughts pertain to residual cell densities, and evaluations of such measures for any intra-operative tissue imaging technology require transparent and reproducible reporting of biopsy location and frequency and how multiple biopsies are handled per patient.

We believe that this is a proof of principle that biological comparisons are a feasible method for validation. Theoretically, fluorescence phantoms might also be used. However, constructing a phantom [3] with the same optical properties of brain tumor or brain tissue is complex and challenging, having to account for factors such as tissue scattering and absorption, autofluorescence, and strong hemoglobin attenuation, which in turn depends on the oxygenation status of hemoglobin and whether hemoglobin is compartmentalized in erythrocytes or not. Furthermore, PpIX is lipophilic and requires surfactants for solubility in order to avoid aggregation in the phantom. Such surfactants might lyse or affect erythrocytes and consequently affect fluorescence [3].

On the other hand, any possible validation will have to ensure long-term reproducibility for longitudinal comparisons. We suspect from our histologically based approach that some of the differences may result from susceptibility to neuropathologist raters. We have therefore included a well-defined cell counting region in addition to the semi-quantitative approach from the past study.

This is the first report which attempts to quantify the performance of a new system for fluorescence detection in comparison with an older established system. We are convinced that in a rapidly evolving field of intra-operative fluorescence detection and resection techniques, establishing such validation procedures will be necessary for testing new hardware, variations in the use of existing drugs, or testing new compounds or drug/device combinations for their performance compared with established standards.

## Limitations

No method for comparing fluorescence visualization devices has been described to date. We aimed to provide the best setting for this objective; however, this method has not been validated before. Therefore, it is a limitation merit mention. A further limitation to our study is that the recorded images under the new filter system are only as good as available recording technology. Thus, additional to the videos, only screenshots can be provided with this article. Despite their good quality, there is a large disparity with what surgeons experienced during surgery, as directly observed through the surgeons’ ocular.

## Conclusion

Maximal safe resection is the well-acknowledged first step of a multimodal therapy algorithm for malignant gliomas. The BLUE 400 AR filter system provides an excellent surgical setting for PpIX fluorescence visualization for safe tumor resection without the need of alternating with white-light microscopy for reorientation, visualization of anatomy, or hemostasis. We believe that with our present investigation, we have demonstrated superior visualization of background while maintaining the same level of biological discrimination of tumor cell infiltration comparing the established method, BLUE 400, with the novel combination.

## Electronic supplementary material

ESM 1 (mov 502 MB)
